# Feasibility of 3D-EIT in identifying lung perfusion defect and V/Q mismatch in a patient with VA-ECMO

**DOI:** 10.1186/s13054-024-04865-8

**Published:** 2024-03-20

**Authors:** Yelin Gao, Ke Zhang, Maokun Li, Siyi Yuan, Qianlin Wang, Yi Chi, Yun Long, Zhanqi Zhao, Huaiwu He

**Affiliations:** 1 State Key Laboratory of Complex Severe and Rare Diseases, Department of Critical Care Medicine, Peking Union Medical College Hospital, Peking Union Medical College, Chinese Academy of Medical Sciences, Beijing, China; 2 Department of Electronic Engineering, Beijing National Research Center for Information Science and Technology (BNRist), Institute of Precision Medicine, Tsinghua University, Beijing, 100084 China; 3 School of Biomedical Engineering, Guangzhou Medical University, Guangzhou, China; 4 Institute of Technical Medicine, Furtwangen University, Villingen-Schwenningen, Germany

## To the Editor,

Three-dimensional (3D) electrical impedance tomography (EIT) is a recently developed lung ventilation and perfusion imaging technique. Conventional two-dimensional (2D) EIT provides information on a single plane, which potentially omits important information concerning changes in global lung conditions. With the advantage of providing lung information on three planes, 3D-EIT may overcome these pitfalls. Recently, the saline bolus method of 3D-EIT has been used to assess regional lung perfusion and ventilation/perfusion (V/Q) mismatch in an animal study [1]. In patients under extracorporeal membrane oxygenation (ECMO) therapy, bedside assessment of lung perfusion remains a major challenge for either 2D or 3D-EIT techniques.

In this study, we demonstrate the feasibility of a novel 3D-EIT method for lung perfusion assessment and its first clinical application in a post-cardiac surgery patient with veno-arterial extracorporeal membrane oxygenation (VA-ECMO) treatment.

## Case presentation

**Fig. 1:**
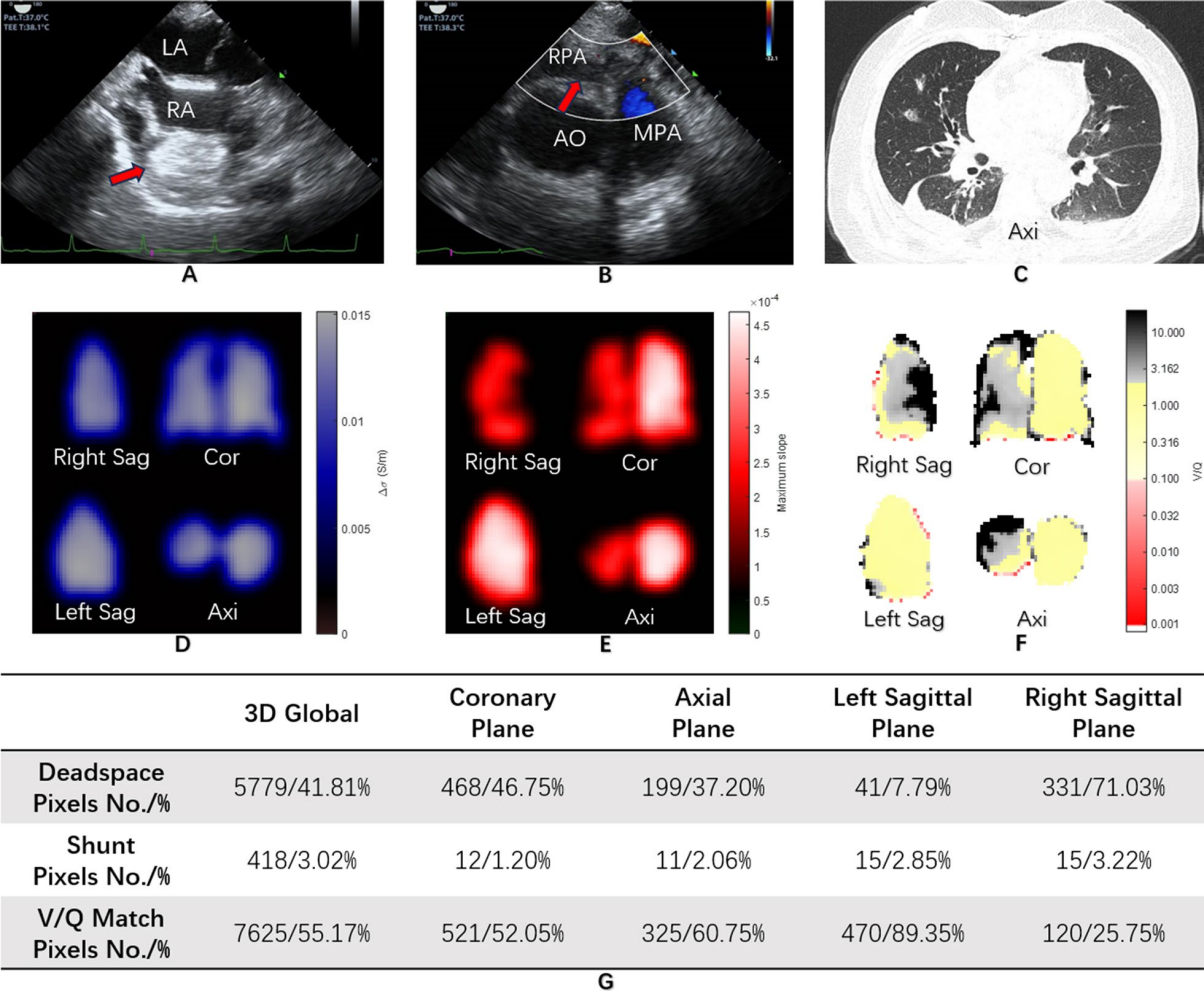
Transesophageal echocardiography (TEE), computed tomography (CT), and saline bolus-based 3D electrical impedance tomography (EIT) results. The EIT measurement system adopted alternating currents with a rms of 2 mA and a frequency of 20 kHz. Voltages were measured at a rate of 20 frames per second. Details of the injection-measurement pattern can be found in [2] (IV-A). The image reconstruction algorithm was run on one NVIDIA Tesla V100 GPU card and the image was produced at a rate of 0.125 frames per second. **A** TEE image of the mid-esophageal bicaval view. The red arrow indicates a massive embolism in the right atrium. **B** TEE image of the mid-esophageal ascending aortic short-axis view. The red arrow demonstrates that the color Doppler found no blood perfusion signal in the right pulmonary artery. **C** Chest CT image indicated that the lung was almost normal. **D** Lung ventilation image of EIT in coronary, sagittal, and axial planes with low-ventilated regions marked in dark blue and high-ventilated regions in light grey. **E** Lung perfusion image of EIT in coronary, sagittal, and axial planes. Regions with low perfusion are displayed in dark red and high perfusion in white. **F** Ventilation/perfusion (V/Q) image of EIT in coronary, sagittal, and axial planes demonstrating the regional V/Q distribution. Regions of dead space (high ventilation and low perfusion) are shown in dark gray. Shunt regions (low ventilation and high perfusion) are marked in red. Areas of matched V/Q ratios are presented in yellow. The cardiac output (2.5 L/min) and minute volume (6 L) were included in the V/Q calculation according to François Perier’s study [3]. **G** The percentage of deadspace, shunt, and V/Q match pixels in 3D global and different planes. *LA* Left atrium, *RA* Right atrium, *RPA* Right pulmonary artery, *AO* Aorta, *MPA* Main pulmonary artery, *Axi* Axial plane, *Cor* Coronary plane, *Right Sag* Right sagittal plane, *Left Sag* Left sagittal plane

A 63-year-old female patient was admitted with multiple space-occupying tumors in the main and right pulmonary artery, which were subsequently confirmed to be intimal sarcoma. During tumor resection surgery, the patient presented with significantly decreased PaO_2_/FiO_2_ ratio and blood pressure and was difficult to wean off cardiopulmonary bypass. Hence, she received peripheral VA-ECMO treatment and was transferred to the ICU for further management. The speed of ECMO was 3500 rpm with blood flow of 3–3.5 L/min. Radiological imaging results are presented in Fig. 1.

## EIT methods

After obtaining informed written consent from the next of kin, 3D-EIT was performed on ICU day 34 for lung assessment. Two belts with 16 surface electrodes each were placed at the level of the 3rd and 5th intercostal spaces. Data were recorded by an EIT device (Infivision 1900, CHINA). Lung perfusion was evaluated by saline bolus-based and pulsatility-based EIT methods. A 20 ml 10% saline bolus was rapidly injected via a central venous catheter during a breathing pause [4]. Images of the pulsatility method were generated based on a separated cardiac-related signal [2].

## EIT results

A significant V/Q mismatch was detected on the right lung with a deadspace of 71.03% in the right sagittal plane and 41.81% in 3D global (Fig. 1D–G). 3D-EIT revealed that ventilation was almost normal across the lung (Fig. 1D). However, regarding perfusion, a severe defect was observed in the right lung (Fig. 1E). With conventional 2D-EIT, abnormal perfusion could also be identified. Dynamic 3D V/Q videos are present in Additional file 1: Video S1, Additional file 2: Video S2 and Additional file 3: Video S3. We extracted data on regional ventilation, perfusion, and V/Q ratios from the coronary, sagittal, and axial planes (Fig. 1D–G).

Comparison of perfusion images using saline bolus and pulsatility methods is shown in Additional file 4: Fig. S1. In the pulsatility strategy, images varied and were more dependent on the slices selected. Whereas the saline bolus-based method provided consistent results of perfusion defect across different planes and slices.

## Discussion

The classical single-belt 2D-EIT has certain pitfalls. It lacks spatial information. Considering the significant inhomogeneity of V/Q distribution in patients under pathologic pulmonary conditions, 2D-EIT fails to capture crucial V/Q information across the lung at coronary and sagittal levels. With more information available, 3D-EIT could potentially provide a more accurate profile for individualized lung assessment. Furthermore, in the single-plane EIT, variations in the sensitive region across the thorax and out-of-plane changes in the conductivity of the volume center could affect the results [1]. Moreover, changes in electrode belt position could significantly influence the result in 2D-EIT, which increases uncertainty when analyzing data between patients. Whereas the double-belt design in 3D-EIT can reduce the impact.

Both saline bolus and pulsatility-based approaches have been adopted to investigate lung perfusion in 2D-EIT. A previous experimental study demonstrated that the saline-based strategy was superior to the pulsatility strategy [5], which is consistent with the present study. For real-time and accurate monitoring of 3D lung perfusion, combining both strategies might be the way forward.

It remains a great challenge for bedside global lung perfusion assessment during VA-ECMO treatment. The flow of VA-ECMO could impact the assessment by directly transferring the injected saline from the femoral vein to the artery [4]. To minimize this effect, we used 20 ml 10% NaCl and lowered ECMO flow to 2L/min during the contrast procedure. A significantly decreased impedance caused by saline injection was observed and the 3D-EIT result was consistent with the clinical judgment and result of TEE.

Yet before the era of 3D-EIT may arrive, certain challenges must be overcome. Firstly, the double-belt design increases inconvenience in clinical procedures. Secondly, the 3D algorithm is complex and time-consuming. With the improvement of hardware and algorithms, real-time bedside 3D-EIT monitoring could be achieved in the near future. Thirdly, further evidence is necessary for continued validation of the efficacy of 3D-EIT and its advantages over 2D-EIT in clinical practice.

In conclusion, 3D-EIT is able to detect lung perfusion defects in a patient with ECMO treatment. It might be a promising new technique for bedside monitoring of V/Q changes in different dimensions.

### Supplementary Information

**Additional file 1: Video S1.** 3D-EIT video in lung perfusion.

**Additional file 2: Video S2.** 3D-EIT video in lung ventilation.

**Additional file 3: Video S3.** 3D-EIT video in Ventilation/perfusion (V/Q) match.

**Additional file 4: Figure S1.** The comparison of saline bolus-based and pulsatility-based methods for accessing lung perfusion. Regions with low perfusion are marked in dark red and high perfusion in white. In the axial plane, lung perfusion images were generated from top to bottom (slices 1 to 20) with an interval of 12mm between each slice. Slice 1 and slice 20 were 23cm apart. The upper and lower belts were at the level of 6 to 7 and 14 to 15 slices respectively. **A** Saline bolus-based electrical impedance tomography (EIT) method in the axial plane. In slices 1 to 2 and 18 to 20, the perfusion signal was vague. **B** Saline bolus-based EIT lung perfusion images in coronary, sagittal, and axial planes. **C** Pulsatility-based EIT lung perfusion images from top to bottom in the axial plane. **D** Pulsatility-based EIT lung perfusion images in coronary, sagittal, and axial planes. The perfusion signal was able to be detected from slices 1 to 18. In slices close to the lung apices and lung base, abnormal perfusion of the right lung could be noted. Yet in other slices, absent perfusion in the right lung cannot be observed. *Axi* Axial plane, *Cor* Coronary plane, *Right Sag* Right sagittal plane, *Left Sag* Left sagittal plane.

## Data Availability

The datasets used and/or analyzed during the current study are available from the corresponding author on reasonable request. The datasets supporting the conclusions of this article are included within the article and its additional files.

## Abbreviations

EIT: Electrical impedance tomography
2D: Two-dimensional
3D: Three-dimensional
V/Q: Ventilation/perfusion
VA-ECMO: Veno-arterial extracorporeal membrane oxygenation
TEE: Transesophageal echocardiography
CT: Computed tomography
