# Supplementary figures and images for: Feasibility of 3D-EIT in identifying lung perfusion defect and V/Q mismatch in a patient with VA-ECMO
Source: Crit Care. 2024 Mar 20;28:90. doi: 10.1186/s13054-024-04865-8 (PMC10956177; doi:10.1186/s13054-024-04865-8)

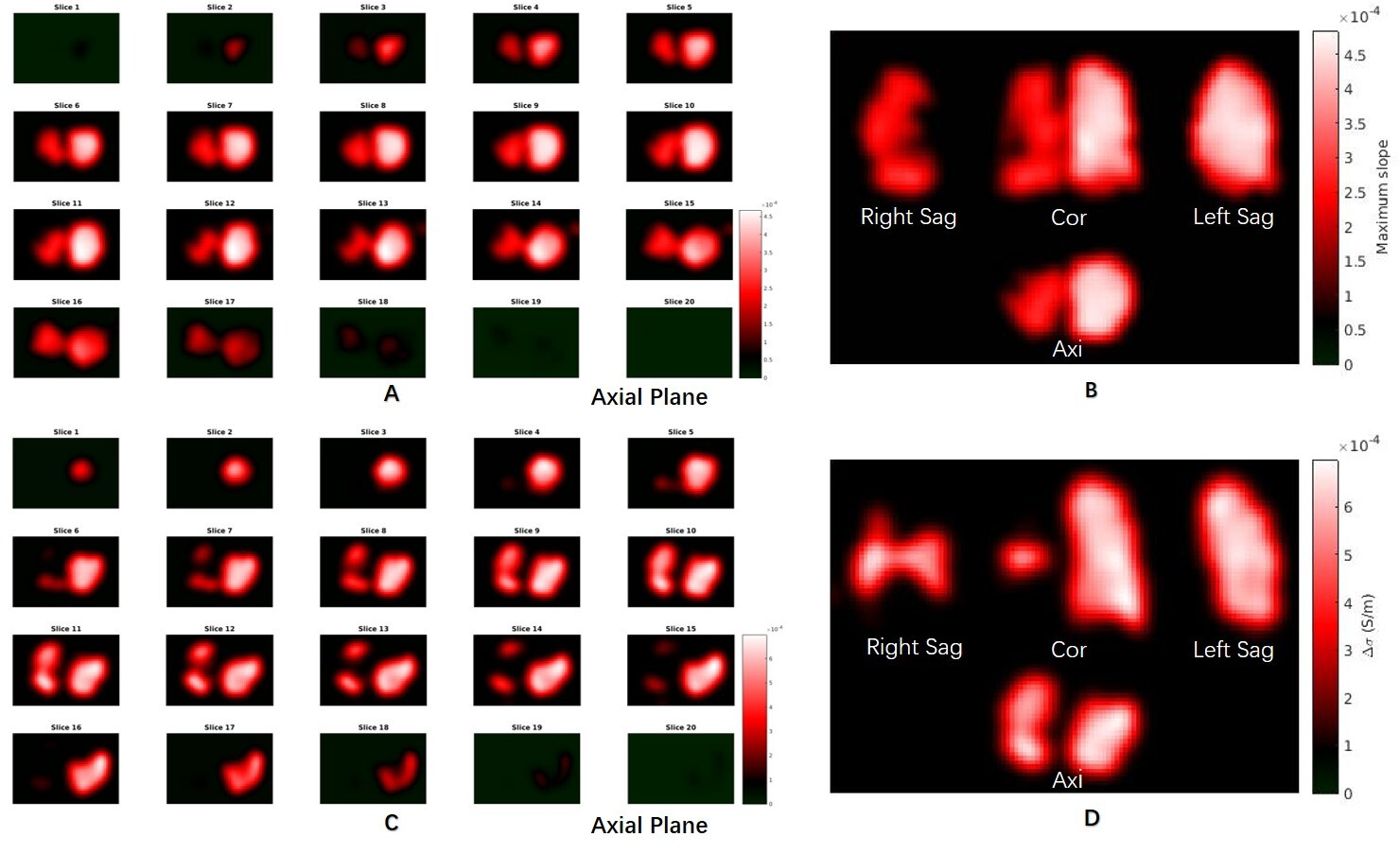

Supplement: Supplementary file 4 — Additional file 4: Figure S1. The comparison of saline bolus-based and pulsatility-based methods for accessing lung perfusion. Regions with low perfusion are marked in dark red and high perfusion in white. In the axial plane, lung perfusion images were generated from top to bottom (slices 1 to 20) with an interval of 12mm between each slice. Slice 1 and slice 20 were 23cm apart. The upper and lower belts were at the level of 6 to 7 and 14 to 15 slices respectively. A Saline bolus-based electrical impedance tomography (EIT) method in the axial plane. In slices 1 to 2 and 18 to 20, the perfusion signal was vague. B Saline bolus-based EIT lung perfusion images in coronary, sagittal, and axial planes. C Pulsatility-based EIT lung perfusion images from top to bottom in the axial plane. D Pulsatility-based EIT lung perfusion images in coronary, sagittal, and axial planes. The perfusion signal was able to be detected from slices 1 to 18. In slices close to the lung apices and lung base, abnormal perfusion of the right lung could be noted. Yet in other slices, absent perfusion in the right lung cannot be observed. Axi Axial plane, Cor Coronary plane, Right Sag Right sagittal plane, Left Sag Left sagittal plane. [file 13054_2024_4865_MOESM4_ESM.tiff]
